# Lithotomy

**Published:** 1872-08

**Authors:** J. T. Happel

**Affiliations:** Memphis, Tennessee


### LITHOTOMY.

BY T. J. HAPPEL, M.D., MEMPHIS, TENNESSEE.

The patient, B. B., a lad aged about eighteen years, was reared in this State. At the age of eight years, he began to be troubled by his urinary organs, at times experiencing great pain in the lumbar regions. Shortly after this, he began to be greatly annoyed by heavy, dragging sensations in the thighs, extending down the leg. He complained also of pain about the penis, and was frequently found pulling at it. The act of micturition was a source of much trouble. The physicians of the neighborhood examined the case, and made various diagnoses. He was, however, treated chiefly for *cystitis* and *stricture!* No sounds had been introduced into his bladder, nor any examinations of his urine made. He gradually grew worse, so that at no time whilst awake was he free from pain.

My attention was called to the case, March 1st, 1872, by Dr. Shackelford, of Trenton, Tennessee, whilst I was stopping in that place. He had seen the case once, and from its history, had suspected a calculus, though, owing to the irritable condition of the bladder, he had not been able to detect it by means of a sound. He requested me to examine him, which I did on the following day. The patient was poorly nourished and very anæmic, though in goood spirits. Questions elicited the facts already stated, together with a statement to the effect that he could at times feel something rolling in his bladder. It was also discovered that urine dripped continually from his penis. An attempt was made to sound him without the use of an anæsthetic; but though the instrument passed quite readily into the bladder, it was at once firmly grasped, the penis becoming at the same time erect, and stopping further proceedings. Chloroform was given after withdrawing the sound, and a second attempt made. The instrument passed in with ease, but the anæsthetic not being very complete, spasm of the muscular fibres of the bladder took place, accompanied by expulsive efforts, causing an evacuation of the contents of the bladder and rectum. Gradually the parts relaxed, and the sounding was proceeded with. For some time, no calculus could be discovered; but just as I was about to withdraw my instrument, it was detected lying low down in the inferior fundus. Some of the urine passed was examined, with the following results: Slightly acid; color paler than usual; specific gravity 10.20; no albumen, except what could be accounted for by the presence of a small quantity of pus.

The patient was sent home, a few miles from Trenton, and put on a course of preparatory treatment. Two days after the examination, he passed much pus per urethra; but this gradually yielded to the free use, internally, of infusion of semina lini. Though he was very feeble, it was decided to operate, there being no other alternative, as his health for some time had been steadily failing. March 9th was fixed for the operation, which was to be the ordinary "lateral incision" of Mr. Erichsen.

At the appointed time, after a second sounding, and the other necessary steps—shaving the perinæum, injecting the bladder, etc.—the bladder was opened, and the stone seized within three minutes. It was then discovered that the calculus was very large. Several vain attempts were made at extraction, the amount of force employed being fully as great as that used in extracting teeth. Finally, after gradually enlarging the opening in the prostate by tearing it, the calculus was brought out. It was of the alternating type, having a lithic acid nucleus, followed by a phosphatic layer, then an oxalate of lime, then another phosphatic one. It measured five and one-third inches in once circumference, by five in the other, and weighed three ounces.

